# Environmental Heat and Renal Health Across Cosmopolitan Populations: A Scoping Review Focused on Sex-Specific Vulnerability

**DOI:** 10.3389/ijph.2026.1609356

**Published:** 2026-03-25

**Authors:** Sung-Hyo Seo, Suwan Go, Yoolwon Jeong

**Affiliations:** 1 Biomedical Research Institute, Gyeongsang National University Hospital, Jinju-si, Republic of Korea; 2 College of Medicine, Dankook University, Cheonan-si, Republic of Korea; 3 Department of Preventive Medicine, College of Medicine, Ewha Womans University, Seoul, Republic of Korea

**Keywords:** heat, renal function, Republic of Korea, scoping review, sex difference

## Abstract

**Objectives:**

Much of the existing evidence on heat–renal associations has focused on Mesoamerican male agricultural workers as the primary high-risk group, leaving sex-specific vulnerability in non-agricultural global populations underexplored. This study synthesized global evidence on heat-related renal outcomes in non-Mesoamerican, non-agricultural populations, with a focus on differences by sex.

**Methods:**

A scoping review was conducted following PRISMA-ScR guidelines. Eligible studies reported sex-stratified renal outcomes and were conducted in non-Mesoamerican, non-agricultural populations. Data were extracted in duplicate and summarized descriptively.

**Results:**

Twenty-one studies from ten countries met the inclusion criteria. Heat exposure was associated with increased risks of acute kidney injury, nephrolithiasis, and urinary tract infections. Men generally showed greater vulnerability to acute kidney injury and stone-related outcomes, whereas women more often demonstrated heat-related increases in urinary tract infections.

**Conclusion:**

Our findings indicate that the risk of heat-related renal morbidity is not confined to the traditionally studied Mesoamerican male agricultural workforce but may also represent an emerging health concern in urban and metropolitan settings. These sex-specific patterns highlight the need for gender-responsive approaches in heat–renal research and public health planning.

## Introduction

Environmental heat exposure has increasingly been recognized as an important determinant of renal distress [1–3]. Much of the accumulated evidence linking heat to renal injury has centered on agricultural populations in Mesoamerica, where chronic dehydration, intense physical exertion, and extreme temperatures have contributed to the emergence of Mesoamerican nephropathy (MeN) or chronic kidney disease of unknown etiology (CKDu) [2–6]. These conditions have been widely regarded as region-specific, occupationally driven disorders, largely affecting male manual laborers. However, emerging studies now suggest that heat-related kidney dysfunction is not confined to this geographic or occupational context. Reports from non-Mesoamerican regions and from general, non-agricultural populations indicate that heat-associated renal impairment may represent a broader and increasingly global public health concern rather than an endemic phenomenon restricted to a single region or workforce [7–9].

Despite the growing recognition of heat-related renal risks, sex-specific patterns have been insufficiently explored. Existing Mesoamerican evidence consistently identifies men as the higher-risk group, reinforcing the view that male agricultural laborers are uniquely susceptible to heat-related kidney injury [3–6]. Yet outside agricultural contexts and beyond the Mesoamerican region, the extent to which gender and sex modifies the relationship between heat exposure and various renal outcomes remains unclear. Biological, occupational, behavioral, and sociocultural factors may differentially influence heat vulnerability among men and women, but these pathways have not been systematically synthesized across global populations [10]. Clarifying sex-specific differences in heat–renal associations is essential for understanding disease mechanisms, identifying at-risk groups, and informing mitigation strategies under accelerating climate change.

The objectives of this scoping review are to provide a comprehensive overview of evidence on environmental heat exposure and renal health beyond the Mesoamerican context. This review focuses on (Population) studies conducted among non-agricultural general adult populations, (Concept) examining the effects of environmental heat exposure on renal health and/or renal diseases, and (Context) with particular attention to studies from non-Mesoamerican regions that report gender-specific differences. Specifically, this review seeks to answer the following research questions:

1. What is the nature and extent of heat–renal associations across non-Mesoamerican settings, particularly among non-agricultural and general populations, and how consistently are these associations reported across studies?
2. Do the health effects of environmental heat exposure differ across renal disease categories—such as acute kidney injury (AKI), chronic kidney disease (CKD), kidney stones, nephritis, and urinary tract infections (UTIs)—and does sex modify these patterns of disease-specific vulnerability?

By addressing these questions, this review aims to understand the global relevance of heat-related renal vulnerability and to inform future research and climate resilience strategies.

## Methods

This study is a scoping review of original research examining the association between extreme heat and renal health, with a particular focus on sex differences. We followed the Preferred Reporting Items for Systematic Reviews and Meta-Analyses extension for Scoping Reviews (PRISMA-ScR) to guide the development and conduct of this review [11]. An *a priori* protocol of this study was registered with the Open Science Framework (OSF; https://osf.io/ts9h5) and is currently under embargo [12]. Ethical approval was not required for this study, as it involved the review of previously published literature, contained no individual human data, and was conducted in full alignment with the Bioethics and Safety Act (Act Number 15188) and the Ordinance of the Ministry of Health and Welfare on the Bioethics and Safety Act (Ordinance number 1048) of the Republic of Korea.

### Search Strategy

Between September 16 and September 30, 2025, we conducted a comprehensive search of three electronic databases: CINAHL, Embase, and PubMed (MEDLINE). The search strategy incorporated combinations of terms aligned with the Population – Concept - Context elements of the review question, including “general population,” “environmental heat exposure,” “renal health,” “renal diseases,” “non-Mesoamerican” settings, and “global” scope. For the MEDLINE search, both Medical Subject Headings (MeSH) and free-text terms were used, while for Embase, Emtree and Emtree-preferred terms were applied alongside keyword searches. The detailed search strategy is provided in Supplementary Material S1. Although sex differences were the primary focus of this review, including sex-related terms in the search strategy did not yield a sufficient number of relevant articles, as studies with sex-stratified analyses often did not explicitly mention this in the title or abstract. Therefore, a broad search without sex-specific keywords was performed, and studies presenting sex-stratified renal outcomes were subsequently identified during the screening process. Given the relatively limited body of evidence on this topic, we also conducted citation tracking and snowball searching to capture additional relevant studies. The search was restricted to articles published after the year 2000, corresponding to the period when Mesoamerican nephropathy began to be recognized in the scientific literature.

### Selection Process and Eligibility Criteria

**FIGURE 1:**
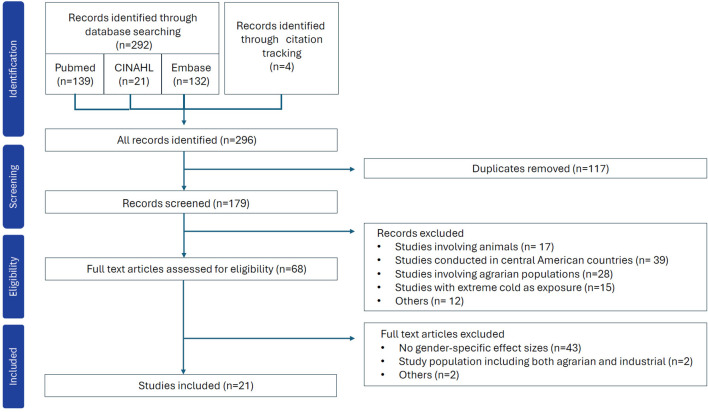
Flowchart outlining the study search and selection process in this scoping review of heat and renal health with a focus on sex-specific vulnerability (South Korea, 2025).

A total of 292 records were identified through database searches and citation tracking. After removing duplicates, 179 records were left for initial screening. The title and abstract screening were conducted independently by two reviewers (YJ and SG), with each record screened in duplicate. Rayyan AI software (http://rayyan.qcri.org) was used for deduplication and for managing the screening process. Any discrepancies were resolved through discussion with a third reviewer (SS). This initial screening procedure was pilot-tested on a subset of 35 studies to ensure consistency in reviewers’ assessments. A total of 111 articles were removed through abstract screening as it did not meet the predefined inclusion criteria, which were studies that 1) involved human participants, 2) assessed exposure to outdoor ambient heat, as well as passive heat exposure, 3) reported renal morbidity outcomes—such as hospital admissions, emergency department visits, or mortality—or physiologic indicators related to renal function, 4) presented sex-specific differences in risk or attributable measures, rather than simple differences in case counts, 5) were conducted in non-Mesoamerican countries, and 6) were published in English. Studies were excluded if they were conducted in central American countries, involved animal subjects, or focused exclusively on agricultural or farming populations. Studies that did not report sex-specific results, thereby preventing assessment of whether environmental heat disproportionately affects renal health by sex, were also excluded. Despite such exclusion criteria, studies from central American countries were included if the study populations were urban or general populations rather than agricultural workers. In accordance with the PRISMA-ScR framework, formal risk of bias assessment and quality appraisal of individual studies were not conducted, as these procedures are not required components of scoping review methodology. A total of 86 articles underwent full-text review, resulting in a final set of 21 studies included in our scoping review [Figure 1].

### Data Extraction and Synthesis

Data extraction was performed independently by two reviewers (YJ and SG) using a pilot-tested extraction template. Extracted information included study title, authors, year of publication, country, study setting, and study population, as well as all variables required to address the elements of the research question—such as definitions and thresholds of heat exposure, renal morbidity and mortality outcomes, physiological biomarkers of renal dysfunction, (e.g., estimated Glomerular Filtration Rate (eGFR) <60 mL/min/1.73 m^2^), and key sex-specific findings, which refer to sex-based differences between males and females as reported in the original studies. Data extraction proceeded in two stages: (1) a pilot and training stage, during which both reviewers extracted data from a subset of studies to ensure consistent application of the extraction template; and (2) a full extraction stage, where both reviewers independently extracted all variables for every included study. Any discrepancies were resolved through discussion, and unresolved disagreements were adjudicated by a third reviewer (SS). Extracted data were summarized descriptively and classified based on the direction and statistical significance of the reported effect estimates. To visualize the findings, a heat–renal outcome matrix was developed, in which associations between environmental heat exposure and renal outcomes were coded as positive, positive but not statistically significant, negative but not statistically significant, or negative. Statistical significance was defined as p < 0.05. The statistical measures used to report the associations varied across studies, including incidence rate ratios (IRR), relative risks (RR), and odds ratios (OR).

## Results

The basic characteristics of the articles included in the review are presented in Table 1. The included studies were conducted in Australia (n = 6), China (n = 3), the Republic of Korea (n = 2), the United States (n = 4), and Germany, Brazil, Taiwan, New Zealand, Switzerland, and Thailand (n = 1 each). Environmental heat exposure was defined using percentile-based thresholds in most studies (n = 10), typically comparing the 90th percentiles of daily mean or maximum temperature to minimum-risk or reference temperatures. Continuous temperature metrics (n = 5), such as per 1 °C increases in daily mean or minimum temperature, were also frequently applied. Fixed thresholds (n = 4), including definitions such as ≥30 °C–35 °C for ≥3 consecutive days or a calculated flexion point (e.g., 28.8 °C), were also used. Renal outcomes fell into two broad categories: (1) renal diseases, including total renal diseases (n = 11), acute kidney injury (n = 11), chronic kidney disease (n = 6), urinary tract infection (n = 7), nephrolithiasis (n = 3), urolithiasis (n = 8), and other renal conditions (n = 5); and (2) physiologic biomarkers of renal dysfunction (n = 4), such as the internationally recognized cutoff for reduced kidney function (e.g., estimated Glomerular Filtration Rate [eGFR] <60 mL/min/1.73 m^2^). Because individual studies often reported more than one renal outcome, these counts represent overlapping categories rather than mutually exclusive classifications. Study populations were drawn from the general population in most studies, with the exception of a few studies that targeted specific groups such as healthy young adult volunteers undergoing controlled passive heating, older institutionalized adults, or employed urban workers.

**TABLE 1:** Country of study, study population, study design, heat exposure metrics, and renal endpoints of studies included in this review (South Korea, 2025).

Author, year	Country	Heat exposure		Primary endpoint	Study population (number)	Study design	Analysis method
		Definition	Thresholds				
Abreu Junior et al. [13]	Brazil	Monthly lowest, mean, and highest temperatures	Continuous measure	Hospital admissions (nephrolithiasis)	General population (12,507)	Retrospective cohort design	Multivariate linear regression
Borg et al [14]	Australia	1 °C increase in daily dry bulb temperature (max, min, average), during warm season	Continuous measure (per 1 °C increase)	Emergency department and hospital admissions (AKI, CKD, UTI, urolithiasis)	General population (83,519)	Ecological time-series design	Negative binomial regression
Borg et al [15]	Australia	3-day daily mean temperature; maximum-temperature thresholds	Heatwave: ≥1 positive excess heat factor day within any 3-day period, severe heatwave: ≥85th percentile	Emergency department and hospital admissions (AKI, CKD, UTI, urolithiasis)	General population (83,519)	Ecological time-series design	Negative binomial regression
Chen et al. [16]	Taiwan	1 °C increase in yearly-averaged wet bulb globe temperature	Continuous measure (per 1 °C increase	Impaired kidney function (eGFR <60 mL/min/1.73 m^2^)	General population (114,483)	Retrospective cross-sectional design	Multivariable logistic regression
Freemas et al [17]	USA	Induced hyperthermia via passive heating	Core temperature elevation of approximately 0.8 °C	Renal vasoconstrictor and vasodilator responses	Healthy young adult volunteers (25)	Block-randomized crossover experimental design	Linear mixed-effects models, pairwise comparisons
Guo et al. [18]	China	Daily mean temperature	97.5th temperature percentiles vs. Minimum mortality temperature	Mortality (total renal diseases, AKI, CKD, urolithiasis, glomerular disease)	Nationwide general population (914,177)	Case-crossover design	Conditional logistic regression + DLNM
Hansen et al. [19]	Australia	Heat waves (≥3 consecutive days of daily maximum temperature ≥35 °C)	Heat waves: ≥3 consecutive days of daily maximum temperature ≥35 °C	Hospital admissions (acute renal failure, renal dialysis)	General population (90,720)	Time-series design	Poisson regression, negative binomial regression
Kim et al. [20]	Republic of Korea	Daily mean ambient temperature	90th and 99th temperature percentiles vs. Minimum morbidity temperature	Emergency department and hospital admissions (AKI, CKD)	General population (1,255,671)	Retrospective cohort design	Quasi-Poisson regression + DLNM
Lim et al [21]	Republic of Korea	Daily mean temperature, during warm season	Flexion point of 28.8 °C	Hospital admissions (AKI)	General population (24,800)	Time-series design	Piecewise linear regression
Lo et al [22]	New Zealand	Monthly mean temperature, hours of sunshine	Continuous measure (per 1 °C increase)	Acute presentation of urolithiasis in hospitals	General population (7.660)	Retrospective cohort design	Poisson regression
Lu et al [23]	Australia	1 °C increase in daily mean temperature, hot season	Continuous measure (per 1 °C increase	Hospital admissions (UTI urolithiasis, renal failure)	General population (238,427)	Case-crossover design	Conditional logistic regression + DLNM
Ogbomo et al. [24]	USA	Daily mean temperature	95th, 97th, or 99th temperature percentiles	Hospital admissions (renal diseases)	General population (18.073)	Case-crossover design	Conditional logistic regression
Qu et al [25]	USA	Daily mean temperature	90th percentile temperature, month- and county-specific	Emergency department admissions (AKI, CKD, UTI, urolithiasis, nephritis/nephrosis)	General population (1,114,322)	Case-crossover design	Conditional logistic regression + DLNM
Schanz et al. [26]	Germany	≥3 consecutive days with maximum temperatures≥30 °C and a heat alarm from the German meteorological service	≥30 °C for ≥3 days vs. indoor/outdoor temperature ≤26 °C (control)	Renal tubular stress urinary biomarker (TIMP-2, IGFBP7)	Older adults with median age 82 (68)	Observational cohort sub-analysis	Multivariate linear regression
Schulte et al. [27]	Switzerland	Daily maximum temperature, during warm season	Moderate heat = 29 °C; extreme heat = 34 °C	Emergency department admissions (AKI, CKD, UTI, urolithiasis)	General population (148.210)	Time-series design	Conditional Poisson regression + DLNM
Talukder et al. [28]	Australia	Daily mean temperature	90th temperature percentiles vs. Minimum-risk temperature	Emergency department admissions (AKI, CKD, UTI, urolithiasis, glomerular disease)	General population (52,057)	Time-series design	Quasi-Poisson regression + DLNM
Tawatsupa et al. [29]	Thailand	Self-reported occupational heat stress	Not applicable (self-reported frequency)	Self-reported, doctor-diagnosed renal disease	General working population (37.816)	Prospective cohort design	Multivariable logistic regression
Vicedo-Cabrera et al. [30]	USA	Maximum daily wet bulb globe temperature	90th and 99th percentile vs. 10 °C reference	Emergency department admissions (nephrolithiasis)	General population (132,597)	Case-crossover design	Quasi-Poisson regression + DLNM
Xu et al [31]	Australia	Hourly ambient temperature	95th percentile (33.6 °C) vs. 50th percentile (26.1 °C)	Emergency department admissions (AKI)	General population (1.815)	Case-crossover design	Conditional logistic regression
Yang et al [32]	China	Daily mean temperature	Daily mean temperature, with 93rd percentile for ≥2 consecutive days	Hospital admissions (urolithiasis)	General population -workers enrolled in employee insurance (23,492)	Time-series design	Quasi-Poisson regression + DLM
Zhang et al [33]	China	Daily mean temperature	90th–97.5th percentiles for ≥2–4 days, city-specific	Rapid kidney function decline (≥3 mL/min/1.73 m^2^/year decline in eGFR	Older adults aged ≥60 (6,450)	Prospective cohort design	Multivariable logistic regression

*AKI*, acute kidne injury; *CKD*, chronic kidney disease; *UTI*, urinary tract infection; *eGFR*, estimated Glomerular Filtration Rate; *DLM*, distributed lag linear modeling; *DLNM*, distributed lag nonlinear modeling; *TIMP-2*, urinary tissue inhibitor of metalloproteinases, *IGFBP7* insulin-like growth factor binding protein.

### Environmental Heat Exposure and Renal Health in Non-Central American Regions – Overall Trends

Across numerous studies included in the review, environmental heat exposure was associated with an increased risk of hospitalization (either inpatient or emergency department visits) for total renal diseases (n = 11) [Table 2]. By specific renal outcomes, acute kidney injury was the condition most consistently identified as being heat-related [14, 15, 19, 20, 23–25, 27–29]. Nephrolithiasis (n = 2) and urolithiasis (n = 8) were also frequently reported as heat-associated [14, 15, 22, 23, 25, 27, 30, 32] Urinary tract infection showed associations in six studies [14, 15, 23, 25, 27, 28]. Chronic kidney disease demonstrated significant associations in only four studies [14, 15, 20, 28], whereas three others reported non-significant findings [18, 25, 27]. Other renal conditions, such as nephritis/nephrosis and glomerular–tubulointerstitial diseases, also did not show significant heat-related associations (data not shown). Although only a limited number of studies evaluated biomarker-based renal outcomes, heat exposure was nevertheless associated with early indicators of renal stress, including elevated urinary tissue inhibitor of metalloproteinase-2 (TIMP-2) and insulin-like growth factor–binding protein 7 (IGFBP7) levels [26], as well as accelerated declines in eGFR [33]. Most included studies assessed short-term, acute effects of heat exposure, commonly using same-day exposure (lag 0) or up to 14 days, while a smaller number of studies examined longer-term or cumulative exposures.

**TABLE 2:** Environmental heat exposure and sex-specific differences in renal disease in studies included in this review (South Korea, 2025).

Author, year	Analyzed lag period (maximum)	Brief description of renal outcome	Gender-specific differences
Abreu Junior et al. [13]	N/A	Higher mean temperature increased nephrolithiasis hospitalizations (R^2^ = 0.284). Hospitalizations were higher in tropical than subtropical cities, and in tropical regions women had a significantly higher proportion of cases than men	• Tropical: men 48.2%, women 51.8% (p = 0.045) • Subtropical: men 52.1%, women 47.9% (p = 0.058
Borg et al [14]	5 days	A 1 °C increase in daily minimum temperature was associated with an increase in daily emergency department admissions for total renal disease (IRR 1.009, 95% CI: 1.006–1.011), acute kidney injury (IRR 1.037, 95% CI: 1.026–1.048), renal failure (IRR 1.030, 95% CI: 1.022–1.039), CKD (IRR 1.017, 95% CI: 1.001–1.033) urolithiasis (IRR 1.015, 95% CI: 1.010–1.020), UTIs (IRR 1.004, 95% CI: 1.000–1.007). No association was observed for pyelonephritis	• Total renal disease: Men: IRR 1.004 (1.000–1.008), Women IRR 1.005 (1.002–1.008) • Urolithiasis: Men IRR 1.003 (0.997–1.009), Women IRR 1.001 (0.991–1.010) • Renal Failure: Men IRR 1.013 (1.002–1.025), Women IRR 1.023 (1.010–1.036) • CKD: Men IRR 1.007 (0.985–1.028), Women IRR 1.000 (0.976–1.024) • UTI: Men IRR 1.004 (0.998–1.010), Women IRR 1.004 (1.001–1.007) • Pyelonephritis: Men IRR 1.011 (0.987–1.036), Women IRR 1.004 (0.994–1.014)
Borg et al [15]	10 days	Excess heat was associated with total urinary disease (1.046; 1.016–1.076 for ED, 1.090; 1.048–1.133 for admissions), urolithiasis (1.106; 1.046–1.169 for ED; admissions increased but not quantified), acute kidney injury (1.416; 1.258–1.594 for ED, 1.335; 1.204–1.480 for admissions), and kidney failure (increased risk reported without numeric estimates)	• Total urinary disease: men (IRR 1.082), women (1.018) • Urolithiasis: men (1.144), women (1.066) • AKI: men (1.458), women (1.353) • CKD: men (1.213), women (1.000)
Chen et al. [16]	Yearly	Long-term heat exposure was associated with increased risk of impaired kidney function (eGFR <60 mL/min/1.73m^2^) in men but decreased risk in women	• Men: 1-year OR 1.096 (95% CI 1.002–1.199), 5-year OR 1.094 (95% CI 1.002–1.195) • Women: 1-year OR 0.872 (95% CI 0.778–0.976), 5-year OR 0.875 (95% CI 0.784–0.977)
Freemas et al [17]	<24 h	Renal vascular control—assessed by changes in segmental artery vascular resistance —did not differ significantly between normothermia and mild passive heat stress	No significant differences were found between younger men and younger women for either renal vasoconstrictor (p = 0.429) or vasodilator (p = 0.204) responses
Guo et al. [18]	6 days	Extreme heat was associated with increased kidney disease mortality (OR 1.06, 95% CI 1.02–1.10). Glomerular diseases demonstrated a statistically significant heat-related risk (OR 1.05, 95% CI 1.01–1.11)	• Total renal diseases: men OR 1.05 (95% CI 1.00–1.10), women OR 1.08 (95% CI 1.02–1.14)
Hansen et al. [19]	N/A	Heat waves were associated with increased hospital admissions for renal disease (IRR 1.10, 95% CI 1.003–1.206) and acute renal failure (IRR 1.255, 95% CI 1.037–1.519). No significant association was observed for dialysis admissions	Overall renal disease admissions increased more strongly and significantly among women, particularly in the very elderly, whereas acute renal failure showed a stronger heat-related increase in men. (A) renal disease (overall): • All ages: men IRR 1.106 (95% CI 0.981–1.247), women IRR 1.088 (95% CI 1.029–1.151) • 15–64 years: men IRR 1.146 (95% CI 0.986–1.333), women IRR 1.098 (95% CI 1.018–1.184) • ≥85 years: men IRR 1.046 (95% CI 0.817–1.340), women IRR 1.218 (95% CI 1.022–1.453) (B) Acute renal failure: Higher risk in men (IRR 1.350, 95% CI 1.049–1.736), sex-specific estimates for women not reported
Kim et al. [20]	7 days	High ambient temperature was associated with increased emergency hospital admissions for genitourinary system renal diseases, acute renal failure in almost all of the districts, with cumulative RRs ranging approximately from 1.20 to 1.42. No association was observed for CKD.	• Genitourinary system: Significant associations in both sexes; higher in women • Renal diseases: Significant associations in both sexes; stronger in women • Acute renal failure: Significant association in males, not significant in women • Chronic kidney disease (N18):No significant heat effect in either men or women
Lim et al [21]	14 days	AKI admissions increased by 23.3% (95% CI: 14.3–33.0) per 1 °C increase in mean temperature above the 28.8 ^∘^C flexion point in the warm season	The effect was stronger in men. Percentage change in the risk of acute kidney injury admissions per 1 °C temperature increase in mean temperature above the 28.8 ^∘^C flexion point in the warm season: Men +28.3%, Women +16.1%. The flexion points were generally lower for men, suggesting greater vulnerability to mild temperature changes
Lo et al [22]	monthly	The number of acute urine calculi presentations was significantly associated with temperature (r = 0.40, P < 0.001) and hours of sunshine (r = 0.59, P < 0.001). For each 1 °C increase in temperature, acute urine calculi presentations increased by 2.8% (95% CI: 1.3%–4.3%). For each 1-h increase in monthly sunshine duration, presentations increased by 0.2% (95% CI: 0.06–0.33)	(A) Sex distribution & correlation with temperature and sunshine • men: temperature (r = 0.39, p = 0.004), sunshine (r = 0.34, p < 0.001) • Women: Temperature (r = 0.21, p = 0.018), sunshine (r = 0.22, p = 0.124) (B) Temperature effect per 1 °C increase (hospital admissions for urine calculi) • men: +3.0% (95% CI 1.34–4.66) • Women: +2.1% (95% CI 0.39–3.87)
Lu et al [23]	10 days	A 1 °C increase in temperature was associated with a 3.3% (95% CI: 2.81–3.87) increase in hospitalizations for urologic diseases (kidney disease, renal failure, UTI, urolithiasis), renal failure (+5.88%, 95% CI: 5.25–6.51), urolithiasis (+4.61%, 95% CI: 4.09–5.14), and urinary tract infection (+2.69%, 95% CI: 2.15–3.22)	• Urologic diseases: men +3.9% (95% CI: 3.4–4.3), women +2.6% (95% CI: 2.2–3.1) • Kidney disease: men +4.81% (95% CI: 4.24–5.38), women +1.97% (95% CI: 1.41–2.54) • Renal failure: men +6.25% (95% CI: 5.60–6.90), women +5.41% (95% CI: 4.75–6.07) • Urolithiasis: men +5.57% (95% CI: 5.03–6.12), women +1.76% (95% CI: 1.19–2.34) • UTI: men +2.02% (95% CI: 1.43–2.61), women +3.06% (95% CI: 2.50–3.62)
Ogbomo et al. [24]	4 days	Extreme heat (97th percentile) was associated with a 19% increase in odds of hospitalization for renal disease (OR = 1.19, 95% CI: 1.03, 1.38)	Association between hospitalization for renal disease: men: OR 1.24 (95% CI 1.01–1.52), women: OR 1.14 (95% CI 0.93–1.40)
Qu et al [25]	6 days	Extreme heat exposure was associated with a 1.7% (95% CI 0.9%–2.5%) to 3.1% (95% CI 2.3%–4.0%) excess rate of emergency department visits for renal diseases across lag days 0–6. By disease subtype, the strongest heat-related increase was observed for acute kidney injury (+16.5%, lag 0, significant). Heat effects were also significant for kidney stones (+8.2%, lag 2), and urinary tract infection (+2.4%, lag 2). The association for chronic kidney disease and nephritis/nephrosis were non-significant	The association of extreme heat and excess rate of emergency department visits was significant in men (+1.9%∼+4.4%) whereas it was not in women. This sex difference was statistically significant
Schanz et al. [26]	N/A	During heat waves, percentage of elevated levels of renal tubular stress biomarkers - urinary [TIMP-2]·[IGFBP7], a predictor for acute kidney injury – was significantly higher (25.0%) compared to control visits (17.7%)	Elevated urinary [TIMP-2]·[IGFBP7] levels during heat waves were more common in men (75.0%) than in women (14.3%) (p < 0.0001). Mean biomarker levels were also significantly higher in men (0.66) compared with women (0.18) (p = 0.0014)
Schulte et al. [27]	7 days	Extreme heat increased emergency hospital admissions for genitourinary diseases (RR 1.12, 95% CI 1.06–1.19). Significant associations were observed for specific conditions: Urinary tract infection (RR 1.17, 95% CI 1.03–1.33), kidney stones (RR 1.33, 95% CI 1.18–1.51), acute kidney injury (RR 2.28, 95% CI 1.83–2.83), dehydration: RR 7.10 (95% CI 5.67–8.90). No significant association was observed for chronic kidney disease (RR 1.05, 95% CI 0.76–1.45)	• Genitourinary diseases: Men RR 1.18 (95% CI 1.09–1.27), Women RR 1.08 (95% CI 0.99–1.18) • Urinary tract infection: Men RR 1.01 (95% CI 0.84–1.18), Women RR 1.36 (95% CI 1.15–1.62) • Kidney stones: Men RR 1.37 (95% CI 1.19–1.59), Women RR 1.22 (95% CI 0.96–1.55) • Acute kidney injury: Men RR 2.67 (95% CI 2.0–3.57), Women RR 1.85 (95% CI 1.29–2.64) • Dehydration: Men RR 5.92 (95% CI 4.14–8.45), Women RR 8.02 (95% CI 6.0–10.71) • Chronic kidney disease: Men RR 0.94 (0.61–1.43), Women RR 1.21 (0.74–1.98)
Talukder et al. [28]	21 days	Hot temperature was associated with increased hospitalizations for overall kidney-related hospitalizations (RR 1.277, 95% CI: 1.149–1.420), chronic kidney disease, RR 1.269 (95% CI: 1.115–1.444), kidney failure (RR 1.252, 95% CI: 1.107–1.416), urinary tract infection (RR 1.395, 95% CI: 1.103–1.765), kidney stone (RR 1.205, 95% CI: 0.648–2.240), Glomerulo-tubular disease (RR 0.870, 95% CI: 0.541–1.399)	In men, hot temperatures showed a significant effect on total hospitalizations only over lag 0–1 day (RR 1.198, 95% CI 1.004–1.431). In contrast, it was significantly associated with increased risks for women with effects lasting beyond 14–21 days (RR 1.26–1.39)
Tawatsupa et al. [29]	Yearly	Self-reported occupational heat stress, particularly when prolonged, was associated with a higher incidence of doctor-diagnosed kidney disease. Overall, 1.1% of participants developed kidney disease	A significant association of heat stress and incident kidney disease cases (“new cases”) was observed in men (Adjusted OR: 1.48; 95% CI: 1.01–2.16) but not in women (Adjusted OR: 0.87; 95% CI: 0.59–1.28). A significant dose-response trend was observed (P for trend = 0.046 in men but not in women In men: • Physical jobs + heat stress: OR 2.57 (95% CI 1.11–5.93) • Office jobs + heat stress: OR 1.26 (95% CI 0.80–1.98) • ≥35 years + prolonged heat + physical work: OR 5.30 (95% CI 1.17–24.13)
Vicedo-Cabrera et al. [30]	10 days	Higher temperature at 99th percentile were associated with an increased risk of emergency department presentations for kidney stones (RR 1.48; 95% CI: 1.37–1.60). Higher vulnerability among older adults and those with pre-existing diseases (diabetes, hypertension, heart failure, CKD)	• 10-day cumulative heat effect Men: RR 1.73 (95% CI 1.56–1.91), Women: RR 1.15 (95% CI 1.01–1.32) • Extreme daily maximum Wet-Bulb temperature (99th percentile) Men: RR 1.12 (95% CI 1.06–1.19), Women: RR 1.05 (95% CI 0.98–1.14)
Xu et al [31]	48 h	Heat effect was associated with acute kidney at lag 0–6 h (OR 1.37, 95% CI: 1.10, 1.71). Heat effects were strongest among adults aged >64 years (OR 2.93, 95% CI 2.01–4.27), particularly those with pre-existing diabetes (OR 2.51, 95% CI 1.91–3.30), hypertension (OR 2.25, 95% CI 1.61–3.15), heart failure (OR 2.21, 95% CI 1.72–2.84), or CKD (OR 2.59, 95% CI 1.89–3.55)	Heat exposure was significantly associated with acute kidney injury in both sexes, but men were more vulnerable (OR 2.48, 95% CI 1.85–3.32)
Yang et al [32]	7 days	Heatwaves were significantly associated with increased hospital admissions for urolithiasis (RR 1.36, 95% CI 1.08–1.71)	The effect of heatwaves on ureteral calculus morbidity was statistically significant in men (AF = 8.50%, 95% CI 3.23–12.60) but was not statistically significant in women
Zhang et al [33]	N/A	Exposure to middle- and high-intensity heat waves was associated with a higher risk of rapid kidney function decline (decrease in eGFR ≥3 mL/min/1.73 m^2^/year (OR 1.23–1.38)	Men showed higher vulnerability (OR 1.42) compared to women (OR 1.18)

*AKI*, acute kidney injury; *CKD*, chronic kidney disease; *UTI*, urinary tract infection; *TIMP-2*, urinary tissue inhibitor of metalloproteinases, *IGFBP7* insulin-like growth factor binding protein, *eGFR*, estimated Glomerular Filtration Rate, *N/A* not applicable, as the study design (e.g., case-control study, sub-analysis of a cohort study) did not involve specification of a lag period.

### Environmental Heat Exposure and Renal Health in Non–Central American Regions – Sex-specific Differences

**FIGURE 2:**
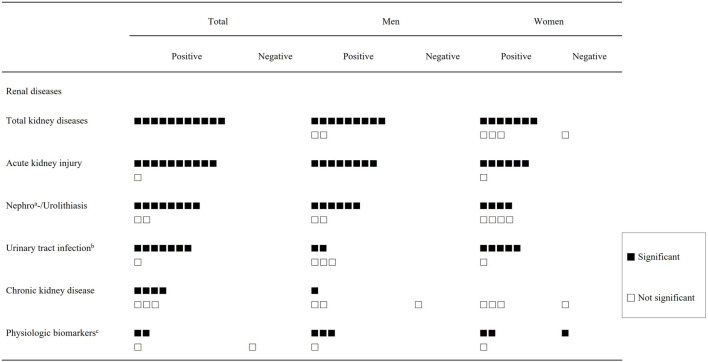
Summary of environmental heat–related renal outcomes by sex (South Korea, 2025). ^a^Two studies, ^b^includes one study with null effect (relative risk = 1.0) among men (Schulte et al. 2024), ^c^Impaired kidney function, kidney function decline, Renal vascular control, Renal tubular stress biomarker.

In renal outcomes that showed significant heat-related associations in the overall population, such as total kidney disease and acute kidney injury, men more frequently demonstrated significant heat-related risks than women [Figure 2]. For total kidney disease, nine studies reported a significant association between heat exposure and increased risk among men [13, 14, 20, 23–25, 27–29], whereas two studies found positive but non-significant associations [18, 19]. In contrast, among women, seven studies reported significant associations [13, 14, 18–20, 23, 28] while four studies reported non-significant findings [24, 25, 27, 29]. A similar pattern was observed for acute kidney injury: eight studies identified significant heat-related increases in AKI risk among men, and none reported non-significant associations. Three studies that identified heat-related increases in AKI risk in both sexes reported stronger associations in men than in women [14, 27, 31].

A male predominance was also observed in nephro- and urolithiasis. Among the studies that reported a significant association between heat exposure and stone-related outcomes, six identified significant associations in men [14, 22, 23, 27, 30, 32] whereas only four did so in women [14, 22, 23, 30]. Three studies found stronger associations in men than in women [14, 22, 23]. No studies reported stronger heat-related associations in women than in men. In contrast, urinary tract infection demonstrated relative female predominance. Only two study reported a significant heat-related association among men [14, 23], whereas five studies identified significant associations among women.

## Discussion

This scoping review identified a consistent trend of increased renal morbidity associated with heat exposure in non–Central American settings, suggesting that the relationship between heat exposure and renal impairment extends beyond the agricultural populations of previously recognized hotspot regions (e.g., Central America, India, Sri Lanka) and may also apply to general populations in cosmopolitan contexts. Although not included in this review, several studies from North America and Europe have demonstrated that high temperatures are associated with increased hospital admissions for renal dysfunction, supporting the relevance of heat–renal pathways in cosmopolitan populations [7–9, 34, 35].

From a physiological perspective, exposure to high environmental heat leads to dehydration, renal vasoconstriction, and elevated urinary osmolarity, resulting in transient reductions in glomerular filtration rate [14, 15]. Repeated episodes of heat-induced acute kidney injury may progress to chronic tubulointerstitial damage and fibrosis over time [15, 19]. Dehydration-related hormonal and metabolic responses (e.g., vasopressin activation, increased fructose metabolism, oxidative stress, urate accumulation) further exacerbate tubular injury, providing a biologically plausible mechanism for heat-related renal injury [19, 25]. Likewise, several studies included in this review demonstrated significant changes in physiological biomarkers—such as reductions in eGFR and increases in tubular stress markers—indicating that subclinical renal injury may occur even in the absence of clinically diagnosed renal disease [26, 33].

The findings of this review on nephro- and urolithiasis align with existing evidence from non-Mesoamerican regions reporting that heat exposure is associated with increased kidney stone events [34]. Existing evidence also suggests regional and seasonal variation within single countries, with elevated risks during periods of higher ambient temperatures and longer sunshine duration, further supporting the role of environmental factors in stone formation [14, 15, 22, 23, 34]. Increased exposure to sunlight is suggested to enhance vitamin D activation and, in turn, intestinal calcium absorption, contributing to hypercalciuria. Simultaneously, high ambient temperatures promote lower urine volume through heat-related dehydration, resulting in higher urinary concentrations of stone-forming solutes such as calcium, oxalate, uric acid, and phosphate [15, 27, 28].

The evidence base for heat–UTI associations remains much smaller than for other renal outcomes such as acute kidney injury or nephrolithiasis. One study included in our review that found an association between UTI risk and extreme heat exposure also reported that this association was transient and limited to lag days 0 through 2, in contrast to acute kidney injury and nephrolithiasis, which tend to exhibit more consistent and cumulative heat-related effects [25]. Age-related effect modification, along with varying definitions of UTI across studies, may contribute to the relatively inconsistent or less robust associations observed for UTIs. From a physiological standpoint, chronic low urine output caused by heat-related dehydration is thought to increase bacterial concentration, elevate urine osmolality and acidity, and reduce the mechanical flushing of bacteria due to decreased urinary volume—offering a plausible explanation for heat-related increases in UTI risk, despite limited and inconsistent empirical evidence [14, 23, 27, 28].

As for CKD, our review found inconsistent associations with heat exposure, and when stratified by sex, only one study demonstrated statistically significant associations in men [15]. Several factors may explain these inconsistent findings. Unlike acute kidney injury or kidney stone events, which typically have short latency periods and are more directly affected by dehydration and heat stress, CKD represents a long-term, multifactorial condition influenced by underlying comorbidities, lifestyle factors, and chronic exposures. One study in our review found that CKD was the only renal category that required a lag period to demonstrate a statistically significant increase in admissions [14]. Consequently, short-term variations in ambient temperature may exert less detectable effects on CKD outcomes, particularly in high-income settings where hydration access, indoor cooling, and healthcare availability can buffer heat-related risks. From a physiological perspective, elevation of vasopressin secretion induced by chronic dehydration may contribute to progressive tubulointerstitial damage and thereby increase susceptibility to CKD, yet more definitive research is needed to clarify the underlying causal mechanisms [14, 15, 36].

This review identified sex-specific differences in the associations between heat exposure and renal outcomes. A greater number of studies reported stronger and statistically significant associations among men for acute kidney injury (8 studies in men vs. 6 in women) and nephrolithiasis/urolithiasis (6 vs. 4, respectively), whereas associations between heat exposure and urinary tract infections were more consistently observed and pronounced among women (5 in women vs. 2 in men). These findings are consistent with previous reports of MeN and CKDu, in which male agricultural workers faced greater risk [4–6, 37], as well as with several non–Mesoamerican studies indicating male susceptibility to heat-related renal events [38, 39]. The result of our study suggest that the risk of heat-related AKI and kidney stone events may extend beyond the traditionally studied Mesoamerican male agricultural workforce and may also represent an emerging health concern for men in global, urban, or metropolitan settings.

Men may be more prone to heat-related AKI due to greater sweat-induced fluid losses and more pronounced heat-driven renal vasoconstriction, all of which can amplify transient reductions in glomerular filtration rate under high-heat conditions [40, 41]. For nephrolithiasis, evidence suggests that men are more likely to rapidly develop calcium and uric acid stones following acute heat exposure, as they exhibit lower urine volume than women during periods of high ambient temperature [15, 30, 32]. Another factor may be that occupational heat exposure and exertional heat stress remain higher in men than in women in many settings, potentially leading to hyperosmolarity, volume depletion, and rhabdomyolysis [42]. Likewise, one study included in our review found that male workers in physically demanding jobs experienced a substantially greater heat-related risk than those in office-based occupations, suggesting that physically intensive work, regardless of whether it involves agriculture, may contribute to heightened susceptibility to heat-related renal outcomes among men [29].

In contrast, studies examining UTIs showed more significant associations among women. Several factors may explain women’s greater susceptibility to heat-related UTI risk. Women experience higher baseline rates of UTI, meaning that additional stressors such as dehydration or reduced urine flow during heat exposure may more readily cause infection [14, 15]. In addition, hormonal influences—particularly lower estrogen levels in postmenopausal women—can reduce the integrity of the urogenital epithelium and alter the vaginal and periurethral microbiota, potentially increasing vulnerability under conditions of heat-related dehydration [14, 27]. This explanation is supported by prior studies showing that heat–UTI associations tend to be stronger among elderly women [43].

Our findings suggest that although MeN and CKDu represent severe and geographically concentrated manifestations of heat-related renal injury, the fundamental biological mechanisms through which heat impairs kidney function are not restricted to these settings. Rather, they appear to operate universally, permitting heat–renal associations to emerge across diverse populations, including those outside traditional high-risk occupational environments. The results further demonstrate discernible sex-specific patterns across renal outcomes, indicating that physiological, occupational, and behavioral differences may jointly modulate susceptibility to heat-related renal dysfunction [44–48].

Despite these insights, substantial uncertainties persist. The disease entities historically described as CKDu or MeN are unlikely to be entirely mutually exclusive from the heat-related renal distress observed in non-Mesoamerican regions included in this review. The heterogeneous clinical manifestations of CKDu and MeN are not fundamentally distinct from the patterns of AKI, nephrolithiasis, and UTI documented in these studies, with the exception of a few contexts in which specific genetic predispositions (e.g., reported in Sri Lanka and India) or particular chemical exposures (e.g., aristolochic acid in Balkan endemic nephropathy) have been identified as major contributing factors [4–6]. Environmental-associated nephropathy is unlikely to arise from a single causal pathway, and the relative contributions of heat exposure and other hypothesized cofactors—such as recurrent dehydration, toxic exposures, infectious agents, water contaminants, and genetic susceptibility—remain incompletely delineated [49–51]. These considerations underscore the need for further investigation into the potential continuum between endemic CKDu and more globally distributed forms of heat-related nephropathy.

In addition, only eight studies explicitly evaluated sex-specific vulnerability by examining the interaction between sex and heat exposure [17, 20, 23–25, 30, 31, 33]. These studies showed mixed findings, with five reporting statistically significant interaction effects, and three reporting no evidence of interaction. The limited use of interaction modelling highlights an important gap in the literature. Although the results of our scoping review suggest a potential heterogeneity between sexes, the evidence yet remains inconclusive, making it difficult to determine whether observed differences reflect true effect modification or variations in study design, population characteristics, or statistical power. Also, differences in lag structures across studies may have influenced both the presence and magnitude of sex-specific associations. Given the diversity in renal outcomes (acute versus chronic) and study designs, heat-related effects may manifest as immediate responses or delayed effects over several days to weeks, and even longer-term cumulative impacts. Such variability in lag structures and outcome definitions may hinder direct comparison of findings across studies. Future studies should incorporate formal interaction analyses along with clearly defined and consistently reported lag structures to better clarify sex-based differences in heat-related renal outcomes.

Future studies should also rigorously adjust for occupational exposures, age, socioeconomic position, climate zone, season, ethnicity, and genetic predisposition; and employ longitudinal or prospective cohort designs capable of elucidating temporal and causal pathways. However, it is worth noting that while occupational exposures may contribute to observed sex differences in heat-related renal outcomes, these differences may not be solely attributable to confounding. Men and women may be differentially exposed due to occupational variations, differentially susceptible to the same level of exposure, or differentially vulnerable in a broader sense [52]. This broader vulnerability encompasses the ability to manage, adjust to, and recover from heat exposure, which can be influenced by contextual factors such as access to protective equipment, work schedule flexibility, hydration resources, and occupational autonomy—factors that may systematically differ between men and women. Future research should consider these multiple dimensions to better understand sex-related vulnerability to heat and inform targeted preventive strategies.

The limitation of this study is the relatively small number of articles that met our inclusion criteria, reflecting the limited volume of existing research on heat–renal associations outside Mesoamerica that also report gender-specific results. In addition, heterogeneity in exposure metrics and outcome definitions may limit the comparability of findings. Definitions of heat exposure and temperature thresholds varied considerably across studies, and few studies comprehensively accounted for confounders such as climate zone, comorbidities, occupational heat exposure, and ambient air pollution. These methodological inconsistencies may limit the ability to directly compare results across studies.

Despite these limitations, a major strength of this review is its focus on sex-specific associations between heat exposure and renal outcomes, an area that has been under-examined in the existing literature. The accelerating trajectory of climate change, which involve rising ambient temperatures, urban heat island effects, and extreme heat events, will likely expose larger segments of the global population to thermal conditions sufficient to induce renal stress. By synthesizing evidence across geographic regions outside Mesoamerica, our study broadens the understanding of heat-related renal vulnerability beyond traditionally recognized hotspot settings.
