# Supplementary material for: Environmental Heat and Renal Health Across Cosmopolitan Populations: A Scoping Review Focused on Sex-Specific Vulnerability
Source: Int J Public Health. 2026 Mar 25;71:1609356. doi: 10.3389/ijph.2026.1609356 (PMC13056881; doi:10.3389/ijph.2026.1609356)
Supplement: Supplementary file 1 [file Supplementaryfile1.docx]

Supplementary file 1. Search strategy applied in this scoping review

**PUBMED**

(("Heat Stress Disorders"[Mesh] OR "Hot Temperature"[Mesh]

OR heatwave*[Title/Abstract] OR "heat wave*"[Title/Abstract]

OR "extreme heat"[Title/Abstract]

OR "ambient temperature"[Title/Abstract]

OR "heat exposure"[Title/Abstract])

AND

("Acute Kidney Injury"[Mesh]

OR "Renal Insufficiency, Chronic"[Mesh]

OR "Urolithiasis"[Mesh] OR "Urinary Tract Infections"[Mesh]

OR AKI[Title/Abstract] OR CKD[Title/Abstract]

OR urolithiasis[Title/Abstract] OR nephrolithiasis[Title/Abstract]

OR "renal failure"[Title/Abstract]))

NOT

(Nicaragua[Title/Abstract] OR Guatemala[Title/Abstract]

OR "El Salvador"[Title/Abstract] OR Honduras[Title/Abstract]

OR Mesoamerica[Title/Abstract] OR "Mesoamerican nephropathy"[Title/Abstract]

OR MeN[Title/Abstract])

AND

(adult[Mesh] OR adult*[Title/Abstract])

NOT

(agriculture[Title/Abstract] OR agricultural[Title/Abstract]

OR farmer*[Title/Abstract] OR crop[Title/Abstract]

OR harvest[Title/Abstract] OR sugarcane[Title/Abstract] OR "sugar cane"[Title/Abstract])

NOT

(perioperative[Title/Abstract] OR intraoperative[Title/Abstract] OR postoperative[Title/Abstract]

OR anesthesia[Title/Abstract] OR anaesthesia[Title/Abstract] OR "warming blanket"[Title/Abstract]

OR "forced air"[Title/Abstract] OR "forced-air"[Title/Abstract] OR "forced air warming"[Title/Abstract] OR "surgical warming"[Title/Abstract] OR "operating room"[Title/Abstract]

OR "operative temperature"[Title/Abstract] OR hypothermia[Title/Abstract]

OR thermoregulation[Title/Abstract] OR "warming device"[Title/Abstract]

OR rewarming[Title/Abstract] OR "cardiopulmonary bypass"[Title/Abstract]

OR CPB[Title/Abstract])

NOT

("vitamin D"[Title/Abstract] OR vitamin-D[Title/Abstract]

OR cholecalciferol[Title/Abstract] OR calcitriol[Title/Abstract])

NOT

(runner[Title/Abstract] OR runners[Title/Abstract]

OR running[Title/Abstract] OR marathon[Title/Abstract] OR ultramarathon[Title/Abstract]

OR exercise[Title/Abstract] OR exercising[Title/Abstract] OR "physical activity"[Title/Abstract]

OR cycling[Title/Abstract] OR cyclist*[Title/Abstract] OR endurance[Title/Abstract]

OR sports[Title/Abstract] OR athlete*[Title/Abstract])

**EMBASE**

(heatwave:ti,ab

OR 'heat wave':ti,ab

OR 'extreme heat':ti,ab

OR 'heat exposure':ti,ab

OR 'ambient temperature':ti,ab)

AND

('acute kidney failure'/exp

OR 'chronic kidney failure'/exp

OR urolithiasis/exp

OR nephrolithiasis:ti,ab

OR urolithiasis:ti,ab

OR AKI:ti,ab

OR CKD:ti,ab

OR 'kidney injury':ti,ab)

NOT

(nicaragua:ti,ab OR guatemala:ti,ab

OR 'el salvador':ti,ab OR honduras:ti,ab

OR mesoamerica:ti,ab OR 'mesoamerican nephropathy':ti,ab

OR men:ti,ab)

AND

(adult:ti,ab OR adults:ti,ab OR 'adult'/exp)

NOT

(agriculture:ti,ab OR agricultural:ti,ab

OR farmer:ti,ab OR farmers:ti,ab

OR crop:ti,ab OR harvest:ti,ab

OR sugarcane:ti,ab OR 'sugar cane':ti,ab)

NOT

(perioperative:ti,ab OR intraoperative:ti,ab

OR postoperative:ti,ab

OR anesthesia:ti,ab OR anaesthesia:ti,ab

OR 'warming blanket':ti,ab

OR 'forced air':ti,ab

OR 'forced air warming':ti,ab

OR 'operating room':ti,ab

OR hypothermia:ti,ab

OR rewarming:ti,ab

OR 'cardiopulmonary bypass':ti,ab

OR CPB:ti,ab

OR 'warming device':ti,ab)

NOT

('vitamin D':ti,ab OR vitamin-D:ti,ab

OR cholecalciferol:ti,ab OR calcitriol:ti,ab)

NOT

(running:ti,ab OR runner:ti,ab OR runners:ti,ab

OR marathon:ti,ab OR ultramarathon:ti,ab

OR exercise:ti,ab OR cycling:ti,ab

OR cyclist:ti,ab OR endurance:ti,ab

OR sports:ti,ab OR athlete:ti,ab)

**CINAHL**

(SU "Heat Stress Disorders"

OR SU "Hot Temperature"

OR TI heatwave* OR AB heatwave*

OR TI "heat wave*" OR AB "heat wave*"

OR TI "extreme heat" OR AB "extreme heat"

OR TI "ambient temperature" OR AB "ambient temperature"

OR TI "heat exposure" OR AB "heat exposure")

AND

(SU "Acute Kidney Injury"

OR SU "Renal Insufficiency, Chronic"

OR SU Urolithiasis

OR TI AKI OR AB AKI

OR TI CKD OR AB CKD

OR TI urolithiasis OR AB urolithiasis

OR TI nephrolithiasis OR AB nephrolithiasis

OR TI "kidney injury" OR AB "kidney injury"

OR TI "renal impairment" OR AB "renal impairment")

NOT

(TI Nicaragua OR AB Nicaragua

OR TI Guatemala OR AB Guatemala

OR TI "El Salvador" OR AB "El Salvador"

OR TI Honduras OR AB Honduras

OR TI Mesoamerica OR AB Mesoamerica

OR TI "Mesoamerican nephropathy" OR AB "Mesoamerican nephropathy"

OR TI MeN OR AB MeN)

NOT

(TI agriculture OR AB agriculture

OR TI agricultural OR AB agricultural

OR TI farmer* OR AB farmer*

OR TI crop OR AB crop

OR TI harvest OR AB harvest

OR TI sugarcane OR AB sugarcane

OR TI "sugar cane" OR AB "sugar cane")

NOT

(TI perioperative OR AB perioperative

OR TI intraoperative OR AB intraoperative

OR TI postoperative OR AB postoperative

OR TI anesthesia OR AB anesthesia

OR TI anaesthesia OR AB anaesthesia

OR TI "warming blanket" OR AB "warming blanket"

OR TI "forced air" OR AB "forced air"

OR TI "forced-air" OR AB "forced-air"

OR TI "forced air warming" OR AB "forced air warming"

OR TI "surgical warming" OR AB "surgical warming"

OR TI "operating room" OR AB "operating room"

OR TI hypothermia OR AB hypothermia

OR TI rewarming OR AB rewarming

OR TI "cardiopulmonary bypass" OR AB "cardiopulmonary bypass"

OR TI CPB OR AB CPB

OR TI "warming device" OR AB "warming device")

NOT

(TI "vitamin D" OR AB "vitamin D"

OR TI vitamin-D OR AB vitamin-D

OR TI cholecalciferol OR AB cholecalciferol

OR TI calcitriol OR AB calcitriol)

NOT

(TI runner* OR AB runner*

OR TI running OR AB running

OR TI marathon OR AB marathon

OR TI ultramarathon OR AB ultramarathon

OR TI exercise OR AB exercise

OR TI exercising OR AB exercising

OR TI "physical activity" OR AB "physical activity"

OR TI cycling OR AB cycling

OR TI cyclist* OR AB cyclist*

OR TI endurance OR AB endurance

OR TI sports OR AB sports

OR TI athlete* OR AB athlete*)
